# Editorial: Promoting Manual Dexterity Recovery After Stroke

**DOI:** 10.3389/fneur.2019.00815

**Published:** 2019-07-30

**Authors:** Martin Lotze, Påvel G. Lindberg

**Affiliations:** ^1^Functional Imaging Unit, Center for Diagnostic Radiology, University of Greifswald, Greifswald, Germany; ^2^INSERM U1266 Institut de Psychiatrie et Neurosciences de Paris, Paris, France

**Keywords:** stroke, neurorehabiliation, recovery, dexterity, prediction

Manual dexterity is often affected following stroke and is a major issue for autonomy in daily living. How best to improve recovery of manual dexterity remains a key clinical and scientific challenge. Although impressive insights in brain plasticity have been demonstrated in the last decade, aging and degree of lesion to the corticospinal tract, along with other factors, severely restrict functional restoration. Individual factors limiting brain plasticity requires further study. Biomarkers of motor recovery and mechanisms of therapy-mediated gains are also issues of research interest.

There have been a number of evidence based therapy approaches which are candidates for further longitudinal studies on motor recovery associated neurophysiological parameters which can then be used as biomarkers/monitoring parameters for therapy control and outcome prediction. For instance, impairment-oriented training, had shown considerable effect sizes for upper limb motor gain following stroke and many neurophysiological parameters have already been identified using a number of different techniques. The contribution of Platz and Lotze in this issue provides an overview on that field and more specifically recent studies on the Arm Ability Training (AAT) approach. AAT incorporates tasks allowing training of various aspects of upper limb sensorimotor control, including selective wrist and finger movements, arm reach and dexterity tasks (manipulation of both large and small objects), and tasks requiring coordination (steadiness and precision). Studies suggest that AAT is superior to conventional therapy, that it can induce sensorimotor learning and that it is coupled with brain plasticity, particularly with recruitment of ipsilesional premotor cortex activation.

When collecting biomarkers, possibly important for prediction of sensorimotor outcome, specific testing of motor impairment is one of the first candidates. Kim et al. reported on a fast version of the Bilateral Arm Reaching Test (BART) for measurement of spontaneous arm use after stroke. This version of BART leads to enhanced reliance on the less-affected arm in stroke patients and the test had good test-retest reliability and correlation with Actual Amount of Use Test (cross-validation). In addition, more comprehensive testing might improve documentation of hand motor impairments for instance including kinematic and kinetic measures such as presented in the review of Collins et al. in this issue. This synthesis showed that stroke was associated with increased movement times, lower velocity, greater trunk displacement, more curved reach-to-path-ratio and reduced movement smoothness. Such measures may serve as targets when developing tailored interventions. Parry et al. emphasize the importance of impaired grasping control and altered grasping configuration after stroke. Interestingly, prehension strategies compound difficulties with grip force scaling and inhibit the synchrony of grasp onset and object release.

With respect to neurophysiology, Zhou et al. investigated the role of electroencephalography (EEG) for the investigation of gains achieved by visuospatial training. Here frontoparietal coherence predicted training-related gains in visuomotor tracking change, measured as change in Success Rate score, highlighting potential importance of sensorimotor connectivity. Cortico (EEG)-Muscular (EMG) coherence measures also enables quantification of motor recovery as shown by Krauth et al. During successful therapy EEG–EMG coherence increased over time, as wrist mobility recovered clinically. Coherence also involved a larger and more bilaterally distributed activation of cortical areas in stroke patients. With respect to Transcranial Magnetic Stimulation, Yarossi et al. focus on the measurement of corticospinal intergrity using TMS. However, here they used motor evoked potential (MEP)-mapping strategies to investigate the responsive areas for eliciting MEPs longitudinally during a motor training in the subacute stage after stroke. They found changes on MEP maps along with changes in motor tests only for responders to motor training both over the lesioned and the non-lesioned hemisphere. They conclude that the association of recovery to bilateral changes in motor topography may depend on integrity of the ipsilesional cortical spinal tract. Rosso and Lamy performed a systematic review of studies correlating upper limb function to resting motor threshold, a TMS measure of functional integrity of corticospinal tract. The findings showed that a low motor threshold correlates with good motor function, both early and in chronic phase post-stroke. Authors concluded that further studies are required on how such TMS measures interact with other factors such as time post-stroke and degree of structural corticospinal damage. Less investigated than MEPs, short intracortical inhibition (SICI) during a motor task is decreased after stroke in the ipsilesional hemisphere and correlated with motor impairment as described here by Ding et al. They concluded, that disinhibition is associated with greater motor impairment and worse dexterity in chronic hemiparetic individuals. This study also highlights benefit of TMS measures when collected during both rest and active states. Finally, neuroimaging measures of structural integrity of corticospinal tract were also found to predict AAT therapy gains in the study by Lotze et al.

Currently, novel interventions are being tested including non-invasive brain stimulation techniques and movement technologies allowing enhanced motivation, intensity of training and enhanced sensory feedback. Interestingly, secondary somatosensory cortex activation is observed especially over the right hemisphere independent on the hand stimulated as reviewed here applying an Activation Likelihood Estimate (ALE) meta-analysis by Lamp et al. This finding suggests a lateralized pattern of somatosensory activation in right secondary somatosensory region. Furthermore, it has also methodological implications for brain mapping studies on the somatosensory system when using a flipping strategy of left or right hemispheric lesions for the purpose to describe functional representation only in an ipsi- and contralesional space.

With this Research Topic we intended to provide latest insight on varied aspects of upper limb and motor and dexterity recovery following stroke. We ended in a range of contributions which have been addressing especially longitudinal observations on stroke survivors, an especially important way to go in the future to understand the neurophysiological basis of motor recovery post-stroke.

## Author Contributions

All authors listed have made a substantial, direct and intellectual contribution to the work, and approved it for publication.

### Conflict of Interest Statement

The authors declare that the research was conducted in the absence of any commercial or financial relationships that could be construed as a potential conflict of interest.

